# Supplementary material for: Complex‐centric proteome profiling by SEC‐SWATH‐MS
Source: Mol Syst Biol. 2019 Jan 14;15(1):e8438. doi: 10.15252/msb.20188438 (PMC6346213; doi:10.15252/msb.20188438)

Ubiquitin E3 ligase (CSN1, CSN8, HRT1, SKP1, SKP2, CUL1, CUL2, CUL3)
Annotated subunits: 8   Subunits with signal: 8
Max. coeluting subunits: 3   Max. completeness: 0.38

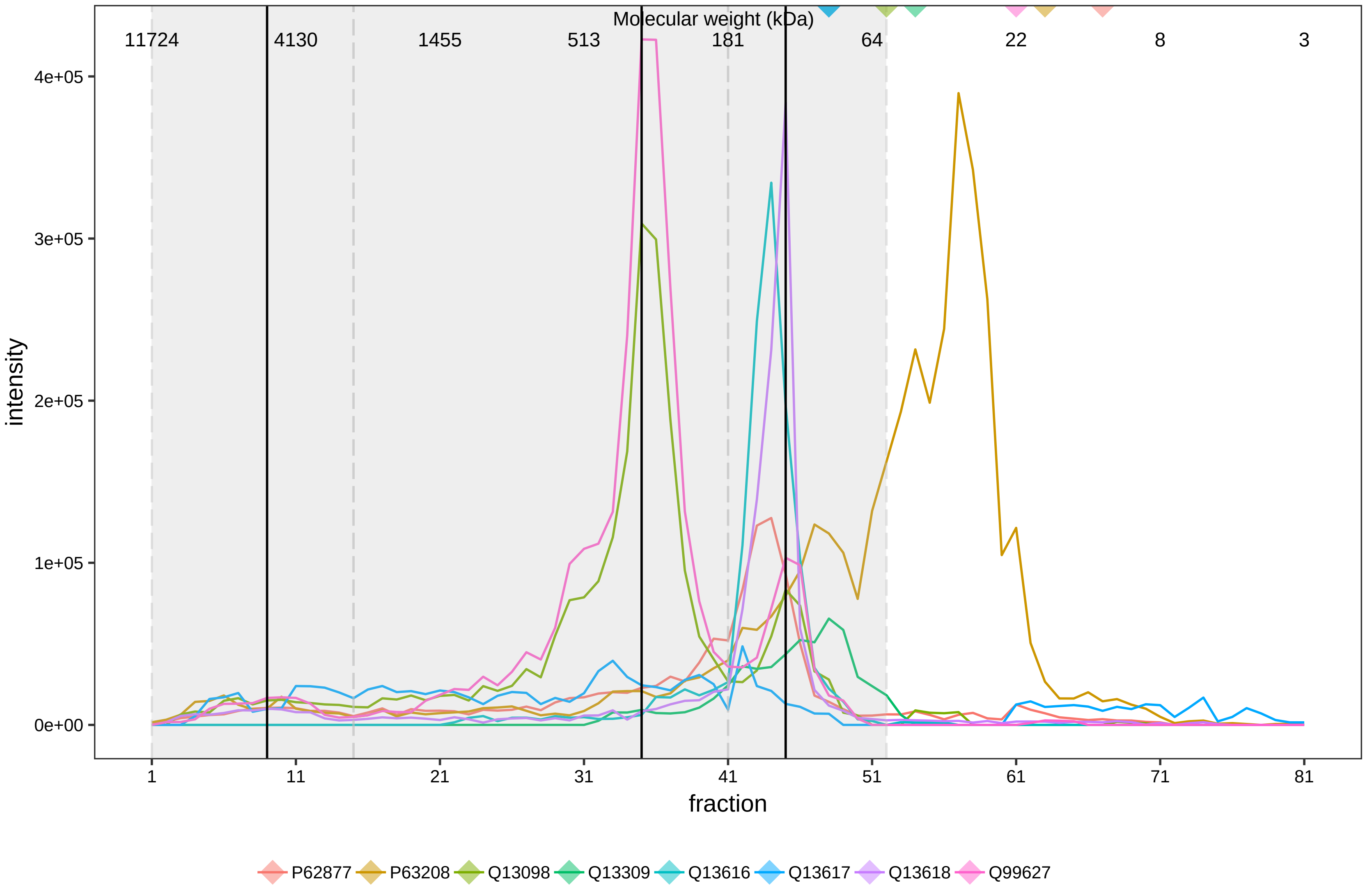

Supplement: Supplementary file 6 — Dataset EV5 [file MSB-15-e8438-s006.zip › feature_plots_corum/2715.pdf]
